# Comparison of intranasal naloxone and intranasal nalmefene in a translational model assessing the impact of synthetic opioid overdose on respiratory depression and cardiac arrest

**DOI:** 10.3389/fpsyt.2024.1399803

**Published:** 2024-06-17

**Authors:** Celine M. Laffont, Prasad Purohit, Nash Delcamp, Ignacio Gonzalez-Garcia, Phil Skolnick

**Affiliations:** ^1^ Research and Development, Indivior, Inc., Richmond, VA, United States; ^2^ Clinical Pharmacology and Pharmacometrics Solutions, Simulations Plus, Buffalo, NY, United States

**Keywords:** synthetic opioids, fentanyl, carfentanil, cardiac arrest, translational model, opioid antagonists, nalmefene, naloxone

## Abstract

**Introduction:**

Using a validated translational model that quantitatively predicts opioid-induced respiratory depression and cardiac arrest, we compared cardiac arrest events caused by synthetic opioids (fentanyl, carfentanil) following rescue by intranasal (IN) administration of the μ-opioid receptor antagonists naloxone and nalmefene.

**Methods:**

This translational model was originally developed by Mann et al. (Clin Pharmacol Ther 2022) to evaluate the effectiveness of intramuscular (IM) naloxone. We initially implemented this model using published codes, reproducing the effects reported by Mann et al. on the incidence of cardiac arrest events following intravenous doses of fentanyl and carfentanil as well as the reduction in cardiac arrest events following a standard 2 mg IM dose of naloxone. We then expanded the model in terms of pharmacokinetic and µ-opioid receptor binding parameters to simulate effects of 4 mg naloxone hydrochloride IN and 3 mg nalmefene hydrochloride IN, both FDA-approved for the treatment of opioid overdose. Model simulations were conducted to quantify the percentage of cardiac arrest in 2000 virtual patients in both the presence and absence of IN antagonist treatment.

**Results:**

Following simulated overdoses with both fentanyl and carfentanil in chronic opioid users, IN nalmefene produced a substantially greater reduction in the incidence of cardiac arrest compared to IN naloxone. For example, following a dose of fentanyl (1.63 mg) producing cardiac arrest in 52.1% (95% confidence interval, 47.3-56.8) of simulated patients, IN nalmefene reduced this rate to 2.2% (1.0-3.8) compared to 19.2% (15.5-23.3) for IN naloxone. Nalmefene also produced large and clinically meaningful reductions in the incidence of cardiac arrests in opioid naïve subjects. Across dosing scenarios, simultaneous administration of four doses of IN naloxone were needed to reduce the percentage of cardiac arrest events to levels that approached those produced by a single dose of IN nalmefene.

**Conclusion:**

Simulations using this validated translational model of opioid overdose demonstrate that a single dose of IN nalmefene produces clinically meaningful reductions in the incidence of cardiac arrest compared to IN naloxone following a synthetic opioid overdose. These findings are especially impactful in an era when >90% of all opioid overdose deaths are linked to synthetic opioids such as fentanyl.

## 1 Introduction

The number of opioid overdose deaths in the United States has continued to increase for more than two decades (1) with modeling studies predicting up to 1.2 million additional fatalities over this decade (2, 3). Illicitly manufactured synthetic opioids (“synthetics”) such as fentanyl have been linked to >90% of the more than 80,000 opioid overdose deaths reported for the 12 months ending in September 2023 (1). Counterfeit pills (produced to resemble medications including methylphenidate, oxycodone, and alprazolam) often containing lethal quantities of synthetics have flooded the United States over the past 5 years (4, 5), and the ready availability of these pills is now the face of what is commonly referred to as the ‘4^th^ wave’ of the opioid epidemic (6). The Drug Enforcement Administration estimated 78.4 million counterfeit pills were seized in 2023, with 70% containing a potentially lethal dose of synthetic opioid (7). It is likely the number of counterfeit pills seized by law enforcement agencies represents only a fraction of the total number smuggled into the United States. Both the sheer quantities and ready availability of these counterfeit pills on the ‘gray market’ often puts unwitting users at a very high risk of a fatal opioid overdose.

As the initial treatment of opioid overdose shifted from the emergency department (8) to first responders (e.g., police, fire department, friends and family of overdose victims), intranasal (IN) naloxone has become the standard rescue agent in a community setting (9). First approved by the FDA in November 2015, IN naloxone (4 mg) can be administered with little or no training, is absorbed as rapidly as an intramuscular (IM) injection (10) and eliminates the potential for needlestick injury. While multiple factors (such as the type and quantity of opioid, route of administration, presence of other drugs, and interval between overdose and intervention) ultimately determine a patient’s prognosis (11), both clinical and preclinical evidence indicate that higher doses of naloxone are needed to reverse a synthetic opioid overdose than are typically used by first responders. Multiple clinical studies have reported high doses of parenteral naloxone (in some cases followed by a naloxone infusion) are required for rescue, with recommendations of up to 12–15 mg if a synthetic like fentanyl is involved (12–14). Moreover, preclinical studies examining respiratory depression in the absence of the multiple factors that complicate interpretation of a clinical overdose have demonstrated that up to ten-fold higher doses of naloxone are needed to reverse respiratory depression produced by fentanyl compared to an opium-based alkaloid like morphine (15) despite comparable affinities at μ-opioid receptors (16). More recent studies (17) have provided key molecular insights which may contribute to this apparent paradox.

Mechanistic modeling studies present an unbiased, alternative approach to estimate the effectiveness of μ-opioid receptor antagonists in a first-responder setting. Model-based approaches have been successfully applied in drug development and can help address information gaps when it is challenging to conduct clinical trials. A translational mechanistic model recently published by Mann et al. (18) was developed to predict the extent of respiratory depression and incidence of cardiac arrest triggered by a synthetic opioid overdose in the absence of antagonist treatment and following administration of IM naloxone. This model was recently implemented to evaluate the efficacy of IN naloxone given as single or multiple doses (19).

In the present study, we leverage and expand this translational model to compare the efficacy of IN nalmefene to IN naloxone, the latter generally considered the “gold standard” for first responders to reverse an opioid overdose. Nalmefene is a more potent μ-opioid receptor antagonist than naloxone and was recently approved for the treatment of opioid overdose (induced by natural or synthetic opioids) in the United States in the form of an IN formulation delivering 3 mg nalmefene hydrochloride (HCl) equivalent to 2.7 mg nalmefene free base.

## 2 Methods

### 2.1 Model overview and implementation

**Figure 1:**
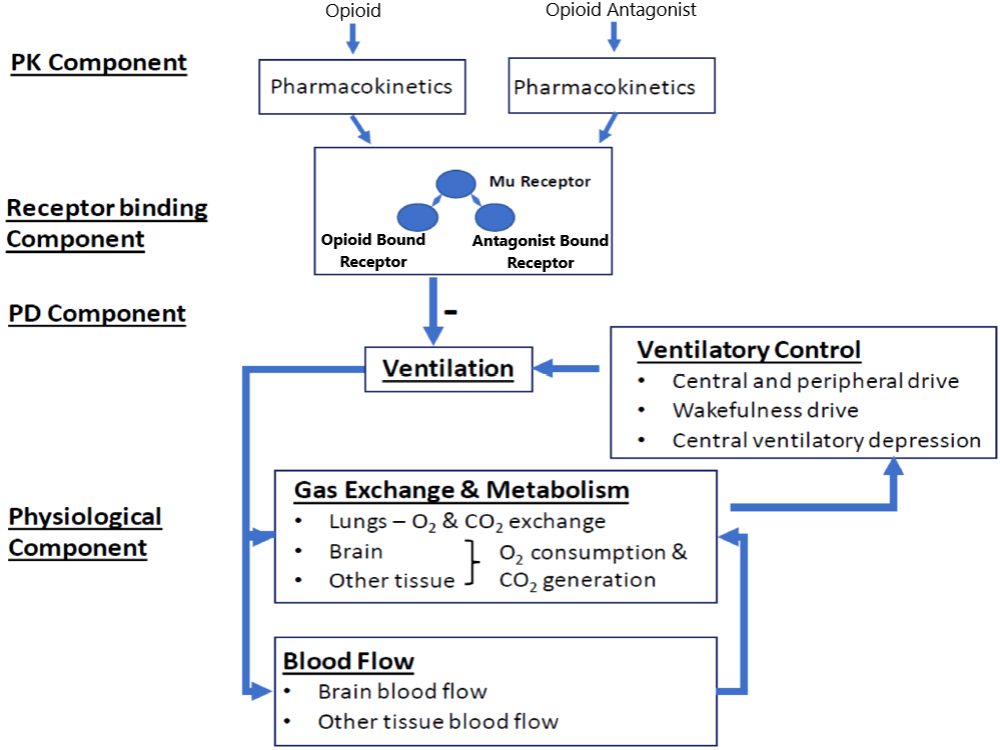
Translational model from Mann et al. (2022). Adapted from Mann et al. publication (18). The model has four components. In the pharmacokinetic (PK) component, compartment PK models convert the doses of opioids and opioid antagonist (e.g., naloxone) through different dosing routes to their free concentrations in the effective compartment. In the receptor binding component, opioid and the opioid antagonist compete to bind to the μ-opioid receptor. In the pharmacodynamic (PD) component, the opioid-bound receptors, but not the opioid antagonist-bound receptors, lead to respiratory depression, through reducing all three ventilatory drives (central chemoreflex, peripheral chemoreflex, and wakefulness drives). The physiological component describes gas (oxygen [O_2_] and carbon dioxide [CO_2_]) exchange and metabolism, ventilatory control, and blood flow control.

We implemented the translational model from Mann et al. (18) (hereafter referred to as the Mann model), which predicts the respiratory depression and incidence of cardiac arrest triggered by a synthetic opioid overdose in the absence of antagonist treatment and following administration of IM naloxone. As shown in **Figure 1**, the model integrates several components describing: 1) the pharmacokinetics of synthetic opioids (given as an intravenous [IV] bolus or infusion) and IM naloxone; 2) the competitive binding between synthetic opioids and naloxone at the μ-opioid receptor; 3) the effects of opioid-bound receptors on ventilation by reducing ventilatory drives (central chemoreflex, peripheral chemoreflex, and wakefulness drives); and 4) the physiological feedback mechanisms involving lung gas exchange, blood gas transport, tissue O_2_ and CO_2_ metabolism, as well as blood flow control.

Mann et al. (18) developed their model based on existing pharmacokinetic and physiologic data as well as experimental data they generated on binding parameters for fentanyl, nine of its derivatives (including carfentanil), naloxone, and buprenorphine. For the physiological and pharmacodynamic components, they leveraged and expanded the work by Ursino et al. (20, 21) using data from animal studies and clinical studies of fentanyl effects on ventilation, both in opioid naïve healthy volunteers and chronic opioid users. Model validation was performed with separate sets of data including *in-vitro* opioid-naloxone competitive binding assays, animal data, and clinical studies of opioid effects on ventilation.

The translational model maintained on a Github public repository (https://github.com/FDA/Mechanistic-PK-PD-Model-to-Rescue-Opioid-Overdose) was cloned to a local computer system and ordinary differential equations coded in C were successfully compiled. Testing confirmed that all interdependent scripts were successfully executed and the simulation results from the Mann et al. publication (18) were successfully reproduced (see **Supplementary Figure 1**).

### 2.2 Pharmacokinetic submodels

Population pharmacokinetic models (nonlinear mixed-effects model) were developed for IN nalmefene and IN naloxone using plasma concentration data from three clinical studies in healthy volunteers: (i) two pharmacokinetic studies published by Crystal et al. (22) assessing a single dose of 3 mg IN nalmefene HCl compared to 1 mg IM nalmefene and two doses of 3 mg IN nalmefene HCl (evaluating two doses in one nostril or one dose in each nostril), and (ii) a pharmacodynamic study published by Ellison et al. (23) comparing the effects of 3 mg IN nalmefene HCl and 4 mg IN naloxone HCl in an experimental model of opioid-induced respiratory depression. Serial blood samples were collected to characterize the pharmacokinetics of both IN nalmefene and IN naloxone in the pharmacodynamic study. Data from the three studies were used to develop the population pharmacokinetic model for IN nalmefene. The population pharmacokinetic model for IN naloxone was developed using concentration data from the pharmacodynamic study. Detailed information on clinical studies and methodology used for population pharmacokinetic analyses are provided in **Supplementary Tables 1**, **2**, respectively.

All clinical studies were conducted in accordance with principles and requirements of the International Council for Harmonization Good Clinical Practice guidelines. Written informed consent was obtained from all participants before starting any study-related procedure. Clinical study protocols, informed consent forms, and all other appropriate study-related documents were reviewed and approved by institutional review boards.

### 2.3 Integration of new components

Ordinary differential equations and parameter estimates from IN nalmefene and IN naloxone pharmacokinetic models were integrated into the Mann model framework. The Mann model utilizes a competitive binding model at the µ-opioid receptor with binding parameters (association rate constant *k_on_*, dissociation rate constant *k_off_*, and steepness parameter *n*) implemented for naloxone, fentanyl, and fentanyl derivatives including carfentanil. A scaling approach was used to estimate nalmefene binding parameters relative to naloxone based on *k_on_* and *k_off_* estimates from Cassel et al. (24). The ratios of nalmefene k_on_ to naloxone k_on_ and of nalmefene k_off_ to naloxone k_off_ using these values were multiplied by the naloxone k_on_ and k_off_ used in the Mann model to obtain scaled binding parameters for nalmefene, retaining the same relative potency. The steepness parameter for nalmefene, *n*, was assumed identical to the one for naloxone (0.86). We also assumed the same plasma to brain equilibration rate constant, *k_e0_*, for nalmefene and naloxone given similar physicochemical properties, using the value of 0.001774 sec^-1^ from the Mann model. **Supplementary Table 3** summarizes pharmacokinetic and binding parameters for naloxone and nalmefene submodels.

### 2.4 Model validation

Model simulations were conducted to assess if the expanded model acceptably reproduced minute ventilation recovery data in the pharmacodynamic study described in Section 2.2 and published by Ellison et al. (23). In this study, subjects breathed a hypercapnic gas mixture (50% oxygen, 43% nitrogen, 7% carbon dioxide) to elevate minute ventilation. Ten minutes after initiating the hypercapnic gas mixture, remifentanil was administered as an IV bolus dose of 0.5 μg/kg followed by an IV infusion at the rate of 0.175 μg/kg/min which was continued for the duration of the study. Fifteen minutes after initiating remifentanil infusion (defined as the nadir of minute ventilation), subjects received either 3 mg IN nalmefene HCl or 4 mg naloxone HCl and were monitored for another 21 minutes.

Simulations were performed in virtual opioid naïve users since the study was conducted with opioid experienced but nondependent healthy volunteers. A single typical subject with mean parameter values generating an “average” profile was simulated for comparison to average measures of ventilation observed in the study. We used the remifentanil population pharmacokinetic model published by Eleveld et al. (25) and remifentanil binding parameters from the Mann model (*k_on_,*8.08E-06 pM^-n^sec^-1^; *k_off_,*2.08E-03 sec^-1^; *n*, 0.70). The plasma to brain equilibration rate constant for remifentanil (*k_e0_*) was fixed to 0.0218 sec^-1^ based on the equilibration half-life of 0.53 minutes reported by Olofsen et al. (26). Remifentanil parameters are summarized in **Supplementary Table 3**.

Modifications of the code were performed to induce a hypercapnic state similar to that observed in the pharmacodynamic study. Specifically, the CO_2_ partial pressure was altered by changing the “P_a_co2” (arterial CO_2_ partial pressure) parameter from 40.28 to 45.30 millimeters of mercury (mm Hg) (delaystates.R script). In addition, to allow the partial pressure of CO_2_ to vary during the simulation, the parameters “P_i_co2” (inspired CO_2_) and “P_a_co2” were changed from 0 to 34 mm Hg and 40 to 45.30 mm Hg, respectively (delaypars.R script).

### 2.5 Opioid overdose simulations

Opioid overdose simulations were conducted with the final model to compare the incidence of cardiac arrest following rescue treatment with IN nalmefene compared to IN or IM naloxone. While the model is capable of simulating multiple physiological outcomes resulting from opioid-induced respiratory depression, Mann et al. (2022) focused on cardiac arrest as an endpoint because in a community setting, the cardiovascular complications produced by asphyxia (the hypoxia and hypercapnia resulting from respiratory depression) are inevitably fatal in the absence of intervention (18, 27). For context, the incidence of opioid-associated out-of-hospital cardiac arrests (OHCA) has been estimated at between 6–14% of all OHCA treated by emergency services personnel (28). However, these estimates generally include only cases that received cardiopulmonary resuscitation, excluding both decedents with a presumed opioid overdose who did not receive cardiopulmonary resuscitation and overdose fatalities where emergency services were not provided (28).

Multiple variables were explored in the simulations, such as the opioid responsible for the overdose (fentanyl or carfentanil), the opioid dose, antagonist dose, and the type of opioid user (chronic vs. naïve). The percentage of subjects with opioid-induced cardiac arrest from a population of 2000 virtual subjects was used as the outcome of interest for each opioid receptor antagonist formulation and dosing regimen. As in the Mann model, cardiac arrest was defined as occurring when the total blood flow reached a value of 0.01 L/min.

For fentanyl and carfentanil, the same IV bolus doses as in the Mann model were simulated to represent “medium” and “high” overdose severities (1.63 mg and 2.97 mg for fentanyl and 0.012 mg and 0.022 mg for carfentanil, respectively). In order to provide additional context for the doses of fentanyl used in these simulations: a 2 mg dose of fentanyl is considered a potentially lethal dose by the DEA (29), with 70% of counterfeit pills seized in 2023 containing a lethal dose (7). The DEA has reported a range of 0.02–5.1 mg of fentanyl per counterfeit tablet (29); an independent report using a very small sample of counterfeit pills reported between 0.6–6.9 mg of fentanyl in a single ‘batch’, confirming the high content variability of illicitly manufactured synthetic opioids (13).

As in the Mann model, a residual minute ventilation volume of 40% of baseline was used as the threshold of respiratory depression to trigger administration of the opioid antagonist (naloxone or nalmefene) which occurred with a 1-minute delay to account for potential time lost for product preparation.

Different dosing scenarios for the opioid antagonist were evaluated, including (i) administration of 1 or 2 doses of IN nalmefene (corresponding to 3 mg and 6 mg of the HCl salt, respectively), (ii) administration of 1, 2, 3, or 4 doses of IN naloxone (corresponding to 4 mg, 8 mg, 12 mg and 16 mg of the HCl salt, respectively), and (iii) administration of IM naloxone using the commercially available 2 mg/2 mL formulation. When multiple doses were simulated for IN naloxone or IN nalmefene, doses were administered simultaneously.

The Mann model describes the application of bootstrapping methods for generating a distribution of *k_on_*, *k_off_*, and *n* estimates for naloxone, fentanyl, and carfentanil. The variability in experimental data and parameter uncertainty in binding parameters were accounted for by using the distributions of binding parameters (N = 2000) generated by Mann et al. (18) and provided in the GitHub public repository. Nalmefene binding parameters were generated based on the distribution of naloxone binding parameters using the scaling approach described in Section 2.3. Consistent with the Mann model, the 2000 sets of pharmacokinetic parameters simulated for nalmefene and naloxone were combined with the 2000 sets of pharmacodynamic binding parameters to generate 2000 virtual subjects.

A bootstrap resampling method was used to estimate the summary statistics (median, and 2.5^th^ and 97.5^th^ percentiles defining a 95% confidence interval) for the incidence of cardiac arrest. A sample with size of 400 was drawn 2500 times from the 2000 virtual subjects simulated for each dosing scenario. The cardiac arrest rate was estimated for each of the 2500 bootstrap samples.

## 3 Results

### 3.1 Pharmacokinetic submodels

Plasma concentrations following IN administration of nalmefene and naloxone were best fitted to 2-compartment models with linear elimination and parallel zero- and first-order absorption with a lag time at the start of the first-order absorption process. The adequacy of the pharmacokinetic models was demonstrated by visual predictive checks showing good concordance between observed plasma concentrations and model predictions (**Supplementary Figures 2**, **3**). Model parameter estimates are displayed in **Table 1** for IN nalmefene and in **Table 2** for IN naloxone. Body weight was identified as a statistically significant covariate on nalmefene apparent clearance (CL/F) with no clinical relevance. Additionally, the model predicted a slower absorption of IN nalmefene in the pharmacodynamic study, with a 35% decrease in first-order absorption rate compared to the two other pharmacokinetic studies also conducted in healthy volunteers. This decrease was attributed to the drying effect of breathing a hypercapnic mixture on the nasal mucosa, which blunted the effects of the nasal absorption enhancer, dodecyl maltoside (23). To reflect the use of IN nalmefene in a rescue setting, absorption parameters estimated in the absence of a mask delivering a hypercapnic gas mixture were used for opioid overdose simulations. The experimental conditions in the pharmacodynamic study did not affect IN absorption of naloxone during the first critical 20 minutes post dose when comparing these data to published data in healthy volunteers (10, 23).

**Table 1:** Final population pharmacokinetic model for intranasal (and intramuscular) nalmefene.

Parameter		Final Parameter Estimate		Magnitude of Variability	
		Population Mean	%RSE	Final Estimate	%RSE
CL/F	Apparent clearance (L/h)	63.7	2.10	15.4%CV	19.3
	Exponent of (WT/74.7) for CL/F	0.572	16.6	–	–
V_c_/F	Apparent volume of distribution, central compartment (L)	15.2	11.8	211%CV	10.3
Q/F	Apparent clearance of distribution (L/h)	81.3	7.23	-	-
V_p_/F	Apparent volume of distribution, peripheral compartment (L)	522	3.05	-	-
INKA	Intranasal first-order absorption rate constant (1/h)	0.497	9.09	39.8%CV	18.4
IMKA	Intramuscular first-order absorption rate constant (1/h)	0.156	5.45	50.4%CV	18.4
D2	Zero-order absorption duration (h)	0.302	7.33	-	-
INFK0	Fraction of intranasal dose with zero-order absorption	0.0485	13.3	-	-
IMFK0	Fraction of intramuscular dose with zero-order absorption	0.0170	10.9	-	-
ALAG1	Lag-time of first-order absorption (h)	0.0615	7.58	-	-
FR	Relative bioavailability for intranasal vs. intramuscular route	0.834	2.23	-	-
STDEFF	Proportional shift in INKA in the pharmacodynamic study	-0.349	17.7	-	-
σ^2^	Residual variability	0.111	4.48	33.3%CV	-

%CV, coefficient of variation expressed as a percentage; %RSE, relative standard error expressed as a percentage; WT, body weight in kg (74.7, median body weight in sample).

**Table 2:** Final population pharmacokinetic model for intranasal naloxone.

Parameter		Final Parameter Estimate		Magnitude of Variability	
		Population Mean	%RSE	Final Estimate	%RSE
CL/F	Apparent clearance (L/h)	396	5.50	39.1%CV	22.4
V_c_/F	Apparent volume of distribution, central compartment (L)	65.7	24.0	240%CV	22.6
Q/F	Apparent clearance of distribution (L/h)	284 (fixed) ^1^	-	-	-
V_p_/F	Apparent volume of distribution, peripheral compartment (L)	102 (fixed) ^1^	-	-	-
KA	First-order absorption rate constant (1/h)	0.998	10.6	-	-
D2	Zero-order absorption duration (h)	0.689	3.67	-	-
FK0	Fraction of dose with zero-order absorption	0.183	23.1	151%CV	30.1
ALAG1	Lag-time of first-order absorption (h)	0.0717	1.82	-	-
σ^2^	Residual variability	0.104	12.2	32.3%CV	-

%CV, coefficient of variation expressed as a percentage; %RSE, relative standard error expressed as a percentage.

^1^Population means of Q/F and V_p_/F were fixed to values estimated by Yassen et al. (30).

### 3.2 Model validation

**Figure 2:**
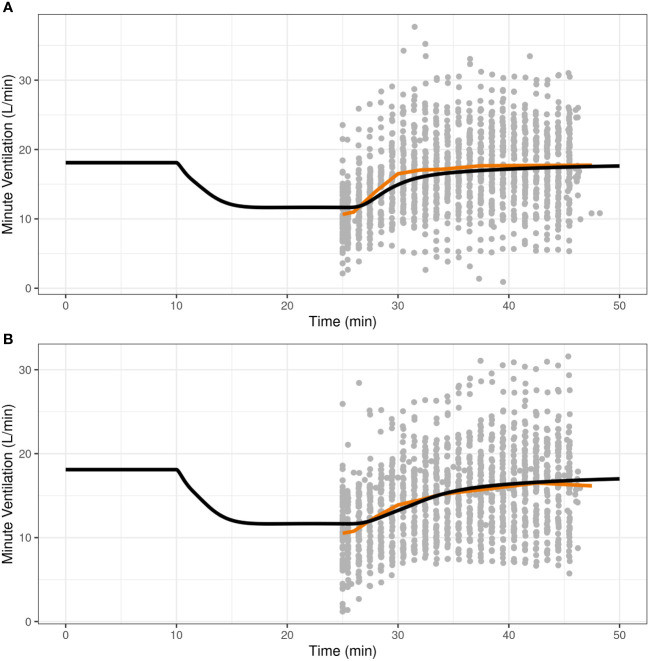
Reproducibility of minute ventilation data in a pharmacodynamic study assessing the effect of intranasal nalmefene **(A)** and intranasal naloxone **(B)** in reversing respiratory depression induced by remifentanil. At time 10 minutes, subjects were administered remifentanil as an intravenous bolus (0.5 μg/kg) followed by an infusion (rate 0.175 μg/kg/min) continuing until the end of the study. At time 25 minutes, subjects received either 3 mg IN nalmefene HCl **(A)** or 4 mg IN naloxone HCl **(B)** to reverse remifentanil-induced respiratory depression. The study was conducted under hypercapnic conditions, with subjects breathing a hypercapnic gas mixture (50% oxygen, 43% nitrogen, 7% carbon dioxide) through a tight-fitting mask. The black curve represents the model predictions for a typical virtual opioid naïve individual, the red line is the average curve based on observed data, and the closed grey circles are the observed values.

Once the translational model was expanded with parameters for IN nalmefene and IN naloxone, the predictive validity of the model was assessed by simulation of the pharmacodynamic study which compared the efficacy of IN nalmefene and IN naloxone in reversing remifentanil-induced respiratory depression (23). Here, simulations accounted for the slower absorption of IN nalmefene resulting from experimental study conditions as described above. As shown in **Figure 2**, the model closely reproduced the change in minute ventilation in the study with minor adjustment to the steepness parameter for remifentanil (changed from 0.70 to 0.75). *No* adjustments were made to either nalmefene or naloxone pharmacokinetic or binding parameters, demonstrating the robustness of the model in describing respiratory outcomes for both opioid antagonists.

### 3.3 Opioid overdose simulations

The model was then applied to compare cardiac arrest outcomes following administration of IN nalmefene and IN or IM naloxone. In virtual chronic opioid users, IN nalmefene resulted in a substantially greater reduction in the incidence of cardiac arrest (**Table 3**). For example, following an IV fentanyl dose of 1.63 mg resulting in cardiac arrest in 52.1% (95% confidence interval: 47.3–56.8) of simulated patients, IN nalmefene 3 mg reduced this rate to 2.2% (1.0–3.8) compared to 19.2% (15.5–23.3) for IN naloxone 4 mg and 29.5% (25.3–34.0) for IM naloxone 2 mg/2 mL. At a higher IV fentanyl dose (2.97 mg) producing cardiac arrest in 77.9% (73.8–81.8) of simulated patients, IN nalmefene 3 mg reduced this rate to 11.6% (8.5–14.8) compared to 47.1% (42.0–52.3) for IN naloxone 4 mg and 54.2% (49.5–59.0) for IM naloxone 2 mg/2 mL. Simultaneous administration of four doses of IN naloxone (4×4 mg) was needed to reduce the incidence of cardiac arrest to values approaching those obtained with a single dose of IN nalmefene, with 3.8% (2.0–5.8) after 1.63 mg fentanyl and 17.0% (13.5–20.8) after 2.97 mg fentanyl (**Table 3**). Two simultaneous doses of IN nalmefene (2×3 mg) further reduced the simulated cardiac arrest percentage to 0.35% (0–1.0) and 3.8% (2.0–5.5) following fentanyl doses of 1.63 mg and 2.97 mg, respectively.

**Table 3:** Simulated incidence of cardiac arrest by opioid, opioid dose, antagonist, antagonist dose and route of administration in chronic opioid users.

	Fentanyl 1.63 mg	Fentanyl 2.97 mg	Carfentanil 0.012 mg	Carfentanil 0.022 mg
No antagonist	52.1% (47.3–56.8)	77.9% (73.8–81.8)	59.2% (54.0–63.8)	90.2% (87.3–93.0)
Intramuscular Simulations				
2 mg/2 mL naloxone^1^	29.5% (25.3–34.0)^2^	54.2% (49.5–59.0)	36.6% (32.0–41.3)	73.7% (69.5–77.8)
Intranasal Simulations				
4 mg naloxone	19.2% (15.5–23.3)	47.1% (42.0–52.3)	27.5% (23.0–31.8)	70.6% (65.8–75.0)
2 × 4 mg naloxone	10.5% (7.5–13.5)	31.8% (27.3–36.5)	15.8% (12.3–19.4)	57.0% (52.0–61.8)
3 × 4 mg naloxone	6.6% (4.3–9.3)	22.6% (18.4–26.8)	10.4% (7.5–13.3)	46.6% (42.0–51.5)
4 × 4 mg naloxone	3.8% (2.0–5.8)	17.0% (13.5–20.8)	6.8% (4.5–9.3)	38.9% (34.0–43.5)
3 mg nalmefene	2.2% (1.0–3.8)	11.6% (8.5–14.8)	3.8% (2.0–5.8)	32.0% (27.5–36.8)
2 × 3 mg nalmefene	0.35% (0–1.0)	3.8% (2.0–5.5)	0.70% (0–1.6)	14.5% (11.0–18.3)

IN, intranasal.

The table shows median (2.5^th^ and 97.5^th^ percentiles) of the cardiac arrest percentage after randomly sampling 400 out of the 2000 virtual subjects 2500 times.

When multiple doses of naloxone or nalmefene were simulated, doses were administered at the same time. Specifically, two, three, and four IN naloxone doses were simulated by administering a dose equal to 8 mg (2 × 4 mg), 12 mg (3 × 4 mg), or 16 mg (4 × 4 mg), respectively. For nalmefene, two IN doses were simulated as 6 mg (2 × 3 mg).

^1^ Generic naloxone formulation, 2 mg/2 mL.

^2^ In Mann et al. publication (18), the estimate for 2 mg/2 mL naloxone was reported as 30% (interquartile range: 28-31%).

In opioid naïve individuals with no pre-existing tolerance to the pharmacological actions of opioids, simulations revealed a higher percentage of cardiac arrest compared to chronic opioid users (**Table 4**). Similar to simulation outcomes in chronic opioid users, IN nalmefene administration resulted in marked reductions in the incidence of cardiac arrest compared to IN naloxone across all dosing scenarios. For example, at a fentanyl dose of 1.63 mg which resulted in cardiac arrest in 74.7% (70.3–78.8) of simulated subjects, IN nalmefene reduced this rate to 7.6% (5.0–10.3) compared to 39.1% (34.3–43.8) for IN naloxone and 48.3% (43.5–53.3) for IM naloxone. As was observed in chronic opioid users, simultaneous administration of four doses of IN naloxone was needed in opioid naïve individuals to reduce the incidence of cardiac arrest to values approaching those obtained with one dose of IN nalmefene (**Table 4**).

**Table 4:** Simulated incidence of cardiac arrest by opioid, opioid dose, antagonist, antagonist dose and route of administration in opioid naïve individuals.

	Fentanyl 1.63 mg	Fentanyl 2.97 mg	Carfentanil 0.012 mg	Carfentanil 0.022 mg
No antagonist	74.7% (70.3–78.8)	90.1% (87.0–93.0)	75.9% (71.5–80.0)	96.4% (94.5–98.0)
Intramuscular Simulations				
2 mg/2 mL naloxone^1^	48.3% (43.5–53.3)	71.8% (67.3–76.0)	56.6% (51.8–61.3)	86.4% (83.0–89.8)
Intranasal Simulations				
4 mg IN naloxone	39.1% (34.3–43.8)	67.6% (63.0–72.0)	50.5% (45.8–55.5)	85.8% (82.3–89.0)
2 × 4 mg IN naloxone	23.8% (19.6–27.8)	53.0% (48.3–57.5)	37.1% (32.3–41.8)	77.1% (73.0–81.3)
3 × 4 mg IN naloxone	16.3% (12.5–19.8)	42.4% (37.8–47.3)	26.8% (22.5–31.3)	69.8% (65.3–74.0)
4 × 4 mg IN naloxone	11.8% (8.8–14.8)	33.4% (28.8–37.8)	20.6% (16.5–24.8)	63.5% (58.8–68.0)
3 mg IN nalmefene	7.6% (5.0–10.3)	25.7% (21.5–30.0)	14.9% (11.5–18.6)	55.0% (50.0–60.0)
2 × 3 mg IN nalmefene	1.7% (0.5–3.0)	10.1% (7.3–13.0)	5.8% (3.8–8.3)	36.8% (32.1–41.8)

IN, intranasal.

The table shows median (2.5^th^ and 97.5^th^ percentiles) of the cardiac arrest percentage after randomly sampling 400 out of the 2000 virtual subjects 2500 times.

When multiple doses of naloxone or nalmefene were simulated, doses were administered at the same time. Specifically, two, three, and four IN naloxone doses were simulated by administering a dose equal to 8 mg (2 × 4 mg), 12 mg (3 × 4 mg), or 16 mg (4 × 4 mg), respectively. For nalmefene, two IN doses were simulated as 6 mg (2 × 3 mg).

^1^ Generic naloxone formulation, 2 mg/2 mL.

**Figure 3:**
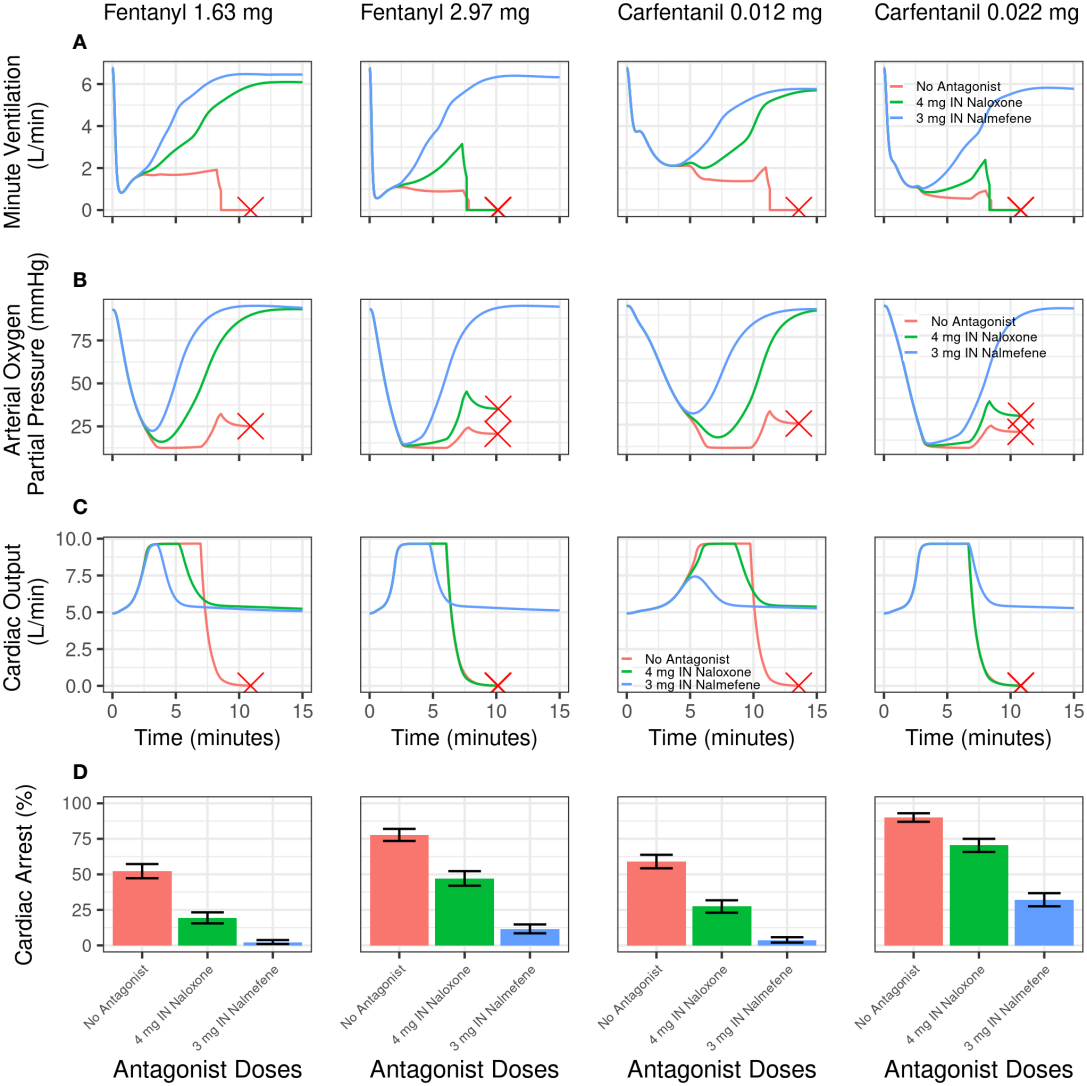
Model simulations evaluating the effect of intranasal nalmefene versus intranasal naloxone on physiological variables and cardiac arrest following an intravenous bolus dose of fentanyl or carfentanil in chronic opioid users. Simulated minute ventilation **(A)**, arterial oxygen partial pressure **(B)**, and cardiac output **(C)** are plotted versus time (in minutes) for a typical virtual subject. The red X designates when a typical virtual subject had a complete cardiac arrest (that is, total blood flow near zero), which stops the simulation. **(D)** shows the simulated percentage of virtual subjects experiencing cardiac arrest. A single dose of IN naloxone 4 mg (green) is compared with a single dose IN nalmefene 3 mg (blue) and no antagonist (red). Error bars represent the 2.5^th^ and 97.5^th^ percentiles after randomly sampling 400 out of the 2000 virtual chronic opioid users 2500 times.

Qualitatively similar outcomes were obtained following IV carfentanil in both chronic opioid users and opioid naïve individuals, although the percentage of simulated cardiac arrests was uniformly higher for carfentanil than for fentanyl because of its much higher affinity for and slower dissociation from μ-opioid receptors (**Tables 3**, **4**). The impact of fentanyl and carfentanil administration on physiological variables (minute ventilation, arterial oxygen partial pressure, and cardiac output) after administration of IN nalmefene, IN naloxone, and no intervention is illustrated in **Figure 3** for a representative chronic opioid user.

## 4 Discussion

There is a compelling body of evidence, both preclinical (15–17) and clinical (12–14, 31), that higher doses of naloxone are needed to reverse a synthetic opioid overdose. Consistent with this evidence, the State Unintentional Drug Overdose Reporting System (SUDORS) database indicates that some form of naloxone had been administered in more than 20% of opioid-related overdose deaths in 2022; in some jurisdictions, more than 35% of decedents had received naloxone (32). Nonetheless, there is resistance to using either higher doses of naloxone or more potent and rapid onset agents like nalmefene (33–35). Much of this resistance is driven by the potential for more severe withdrawal in patients with a pre-existing opioid use disorder and justified by reports that “standard doses of naloxone” needed to reverse an overdose have not risen significantly (36–38) despite the demonstrable rise in synthetic opioids in the illicit drug supply (39). However, conclusions based on anecdotal observations, case studies, and chart reviews suffer from the inherent heterogeneity of an opioid overdose, variability in reporting outcomes, and study quality. For example, multiple reports used to support the position that naloxone doses have not increased attempted to standardize IN, IV, and IM naloxone dosing as “naloxone IV equivalents” based on bioavailability (36–38). While the attempt to standardize naloxone dosing based on plasma exposure is understandable, it does *not* account for differences in early plasma concentrations across routes of administration (18, 19, 40), which is essential for the successful reversal of a synthetic opioid overdose (11, 18, 19). These reports may also introduce a significant reporting bias, with reported EMS rescue rates of ≥99% (38, 41) which underestimate opioid overdose deaths reported in the SUDORS database. A translational model of opioid overdose offers an unbiased approach to estimating the effectiveness of naloxone in the face of a potentially lethal opioid overdose.

Among the most compelling evidence that standard IM doses of naloxone (2 mg/2 mL) favored by many first responders would result in a significant loss of life following an IV synthetic opioid overdose is the translational model developed by Mann et al. (18). These authors evaluated the effectiveness of IM naloxone in reversing hypoxia-induced cardiac arrest produced by fentanyl and carfentanil. Fentanyl doses of 1.63 mg and 2.97 mg were modeled because these doses result in plasma concentrations similar to the mean and one standard deviation above the mean plasma concentrations, respectively, measured post-mortem in a study of approximately 500 fatal fentanyl overdoses (18). In the Mann model, an IV bolus of 1.63 mg fentanyl produced cardiac arrest in approximately 52% of 2000 virtual chronic opioid users in the absence of antagonist treatment. Following IM administration of naloxone (2 mg/2 mL), the incidence of cardiac arrest was reduced to 30%, corresponding to a rescue rate of 42%. The Mann model predicts that IM naloxone will be even less effective at reducing the incidence of cardiac arrest following administration of either 2.97 mg fentanyl (rescue rate of 30%) or the ultrapotent synthetic opioid, carfentanil (rescue rates of 18% to 38% depending on the carfentanil dose: 0.022 mg or 0.012 mg, respectively) (**Table 3**).

Because of the widespread use of IN naloxone in a community setting (9), we implemented and expanded the Mann model to compare the effectiveness of IN nalmefene to standard doses of IN naloxone HCl (4 mg), the latter generally considered the “gold standard” for first responders (22). The model predicts large and clinically significant reductions in the incidence of cardiac arrest following rescue with IN nalmefene compared to IN naloxone under each of four conditions (i.e., medium and high doses of fentanyl and carfentanil) and for each population investigated (chronic opioid users and opioid naïve individuals) (**Tables 3**, **4**). Following a simulated IV overdose with 1.63 mg fentanyl producing cardiac arrest in approximately 52% of patients in the absence of antagonist treatment, the incidence of cardiac arrest following IN nalmefene was 2.2% (rescue rate of >95%) compared to 19.2% following IN naloxone (rescue rate of 63%). Large differences in the effectiveness of IN nalmefene compared to IN naloxone were also evident in overdoses simulating a higher fentanyl dose as well as both the medium and high doses of carfentanil.

The “medium” and “high” doses of fentanyl used in the simulations were based on data from approximately 500 cases of fatal fentanyl overdoses, the great majority with a history of opioid use and/or chronic pain (18). Tolerance to the respiratory effects of opioids is known to develop following repeated use (42), and the incidence of cardiac arrest in this population would be predicted to be lower than those in an opioid naïve population. Implementing the model in opioid naïve individuals confirmed this prediction, with a higher incidence of cardiac arrest following both fentanyl and carfentanil administration compared to simulations in an opioid-tolerant population. Nonetheless, the differences in effectiveness between nalmefene and naloxone were as robust in this population as in simulated chronic opioid users. This is critical because the increased availability of synthetic opioids in counterfeit pills (7) has fundamentally changed the demographics of overdose, with increased numbers of opioid naïve individuals, especially adolescents (43) exposed to potentially lethal doses of synthetic opioids.

Because our simulations demonstrated large reductions in the incidence of cardiac arrest after a single dose of IN nalmefene compared to a single dose of IN naloxone, we examined the rates of cardiac arrest following administration of multiple doses of naloxone. Simultaneous dosing of two, three and four doses of naloxone (total amount of 8, 12, and 16 mg) produced dose-related reductions in the incidence of cardiac arrest. However, at both medium and high doses of fentanyl or carfentanil, four doses of IN naloxone (16 mg) administered simultaneously were needed to reduce the incidence of cardiac arrest to values approaching a single dose of nalmefene (**Tables 3**, **4**). The simulation results for IN naloxone were consistent with recent data by Strauss et al. (19) who used the same translational model as in the present work despite different data used for their IN naloxone pharmacokinetic model. In their simulations, Strauss et al. evaluated the use of either ‘rapid’ or ‘standard’ dosing regimens of IN naloxone and showed that four doses of naloxone given at 2.5-minute intervals would not produce a “meaningfully lower” incidence in cardiac arrest compared to a single IN naloxone dose. They also showed that the simultaneous delivery of two doses of IN naloxone produced additional reductions in the incidence of cardiac arrest compared to one dose of IN naloxone, which drove our decision to evaluate simultaneous (and not sequential) doses of naloxone for comparison with IN nalmefene.

In the Mann model, μ-opioid receptor antagonists were administered one minute after ventilation was decreased to 40% of baseline to mimic a delay between recognizing respiratory depression and administering the reversal agent. In practice, recognizing the signs of an opioid overdose requires training, and there is very often a longer delay in administering the reversal agent. Therefore, with the assumption that an opioid overdose would be immediately recognized and treated, our model predictions likely overestimate the effectiveness of both μ-opioid receptor antagonists in a field setting. Strauss et al. (19) reported that even a brief (3–10 min) delay in administration of IN naloxone could result in a dramatic loss in the effectiveness of IN naloxone. Also, while fentanyl remains the predominant synthetic found in DEA seizures (11), the illicit drug supply varies over time and by region. Thus, the quantities of fentanyl used in the Mann model may not reflect either the actual quantities or composition of opioids (including other opioids such as heroin) in a real-world scenario.

Other limitations of this work are inherent to the translational model itself. Mann et al. used an extensive set of data to develop, calibrate and validate their model, including *in-vitro* binding experiments, pharmacokinetic data, clinical studies of opioid effects on ventilation in chronic opioid users and opioid naïve subjects, and animal studies with severe hypoxia-induced cardiac arrest which could not ethically be conducted in humans (18). As pointed out by the authors, although some datapoints in their validation datasets had high variability, the ratio of mean values between model predictions and observations had a median of 0.95 (interquartile range, 0.90–1.01) across validation datasets, demonstrating a very good performance of the model. One model limitation is the lack of robust pharmacokinetic model for IV carfentanil. Mann et al. (18) had to adjust the pharmacokinetic model for IV fentanyl to predict carfentanil pharmacokinetics and match the carfentanil half-life of 45 minutes reported in the literature. Another limitation is the use of the same pharmacodynamic parameters for all synthetic opioids to describe their effects on ventilatory drives based on receptor occupancy, ignoring possible differences in terms of G protein activation and tolerance mechanisms. Nonetheless, Mann et al. were able to successfully predict the respiratory effects of fentanyl, remifentanil and alfentanil in clinical laboratory studies (18). With respect to the model expansion with IN naloxone and IN nalmefene data, robust pharmacokinetic models were developed from pharmacokinetic data collected in three studies (22, 23). Since the Mann model did not include binding parameters for nalmefene, those were derived from published data (24) using a scaling approach. Additional validation showed that the model was able to closely reproduce the effects of IN nalmefene and IN naloxone in reversing remifentanil-induced respiratory depression in healthy volunteers (**Figure 2**).

Overall, the difference in the apparent effectiveness between IN nalmefene and IN naloxone is consistent with preclinical and clinical evidence including: a higher affinity of nalmefene at μ-opioid receptors (24, 44), higher plasma concentrations of nalmefene delivered at the critical early time points (e.g., 5 min) after dosing (10, 22, 23), and a more rapid onset of action as demonstrated in a clinical model of opioid-induced respiratory depression (23). The model may be further used to assess the effects of other synthetic opioids, including novel synthetic opioids (NSOs) such as the benzimidazoles (e.g., etonitazene and isotonitazene). Because this latter class of synthetic opioids was never approved for human use, additional clinical pharmacology studies (including pharmacokinetic and medical toxicology data [e.g. plasma concentrations from overdose victims]) would be required to adequately interrogate the model.

Nalmefene has a significantly longer plasma half-life (t_1/2_ 7.1–11 h) (22, 45) than naloxone (t_1/2_ ~2 h) (10) as well as higher affinity at μ-opioid receptors (24) which have the potential risk to produce a more severe and longer precipitated withdrawal in individuals with an opioid use disorder. A double-blind, randomized clinical study (46) comparing IV doses of naloxone (2 mg) and nalmefene (1 or 2 mg) in 176 patients admitted to nine emergency departments with suspected narcotic overdose reported that both opioid antagonists produced rapid and robust reversals of respiratory depression in patients with a confirmed opioid overdose. Adverse events were noted in opioid-positive patients in all three treatment arms, but the overall difference among treatment arms was not significant (p > 0.27) and no significant overall time-treatment interactions (measured out to 240 min post-dosing) emerged; no statistical differences in withdrawal outcomes were seen between treatment groups (46). While it is difficult to extrapolate the results of a study conducted in an emergency department to the use of IN naloxone by first responders in a community setting, it is noteworthy that the overall incidence of adverse events following IV administration of these opioid antagonists (46) is lower than in a contemporary study using information provided by first responders and community-based organizations following rescue with 4 mg IN naloxone (47). Furthermore, the higher plasma naloxone concentrations which may be needed to reverse a synthetic opioid overdose also carries an increased risk of precipitated withdrawal. Ironically, multiple doses of IN naloxone administered using a standard regimen (i.e., a dose administered every 2–5 minutes if there is no response) increases the risk of sustained precipitated withdrawal without conferring significant therapeutic advantage compared to a single IN dose (19).

Whether the risks of underdosing with an opioid antagonist outweigh the risks associated with precipitated withdrawal remains a matter of debate. Although the majority of opioid overdoses are not fatal (11), every overdose is potentially lethal in the absence of intervention and the primary goal of opioid antagonist treatment is to prevent death. While withdrawal symptoms precipitated by an opioid antagonist can be unpleasant and distressing, they are medically manageable and rarely life threatening (8). As such, they should not limit the use of more effective reversal strategies, especially when the odds of a synthetic opioid overdose are high (18, 48, 49). On the other hand, there are concerns that precipitated withdrawal could pose acute risks to the patient and medical personnel and could trigger further opioid use to counter the effects of the opioid antagonist (33, 35, 41, 50, 51). Additionally, the knowledge and avoidance of precipitated withdrawal could reduce both use and acceptance of the reversal agent among patients with an opioid use disorder (33, 35, 41, 50). Nonetheless, a recent study (52) of 1152 patients entering treatment for opioid use disorder across 49 addiction treatment facilities reported that most respondents had either no preference (48.4%) or preferred a higher dose (35.9%) reversal agent if they were to experience another overdose. These findings indicate that while the concerns about precipitated withdrawal are valid, the majority of people who have had a lived experience accept the need for effective reversal agents for themselves and others within a community setting (52).

In conclusion, simulations using a validated translational model of opioid overdose demonstrate that a single dose of IN nalmefene produces clinically meaningful reductions in the incidence of cardiac arrest compared to naloxone tools (IN, IM) frequently used by first-responders across a variety of scenarios involving fentanyl and carfentanil. These findings are consistent with converging lines of pharmacological evidence that the rapid delivery of high concentrations of a potent reversal agent favors a successful rescue in the era of synthetic opioids.

## Data availability statement

The raw data supporting the conclusions of this article will be made available by the authors, without undue reservation.

## Ethics statement

The studies involving humans were approved by WCG IRB, 1019 39th Ave. SE, Suite 120, Puyallup, WA 98374 and IntegReview Institutional Review Board, 3815 S Capital of Texas Hwy, Austin, TX 78704. The studies were conducted in accordance with the local legislation and institutional requirements. The participants provided their written informed consent to participate in this study.

## Author contributions

CL: Conceptualization, Supervision, Writing – original draft. PP: Supervision, Writing – review & editing. ND: Formal analysis, Writing – review & editing. IG-G: Formal analysis, Writing – review & editing. PS: Conceptualization, Supervision, Writing – original draft.
