# Supplementary material for: Comparison of intranasal naloxone and intranasal nalmefene in a translational model assessing the impact of synthetic opioid overdose on respiratory depression and cardiac arrest
Source: Front Psychiatry. 2024 Jun 17;15:1399803. doi: 10.3389/fpsyt.2024.1399803 (PMC11215134; doi:10.3389/fpsyt.2024.1399803)
Supplement: Supplementary file 1 [file DataSheet_1.pdf]

Supplemental Material

Comparison of Intranasal Naloxone and Intranasal Nalmefene in a Translational Model Assessing the Impact of Synthetic Opioid Overdose on Respiratory Depression and Cardiac Arrest

Celine M. Laffont[1*], Prasad Purohit[1], Nash Delcamp[2], Ignacio Gonzalez-Garcia[2] and Phil Skolnick[1]

[1] Research and Development, Indivior, Inc., Richmond, VA. 23235

[2]Clinical Pharmacology and Pharmacometrics Solutions, Simulations Plus, Buffalo, NY 14024

*** Correspondence:**
Corresponding Author
celine.laffont@indivior.com

Table S1 Clinical Studies Used for Population Pharmacokinetic Modeling and Validation of the Translational Model

Study (NLM identifier; citation)	Study Design	Dose/Regimens	Subjects
PK Study 1 (NCT04759768; Crystal et al., 2024)	Open-label, randomized, 2-period, 2-treatment, 2-sequence, crossover study to determine the PK and safety of one dose of 3 mg nalmefene HCl IN spray compared to one dose of 1.0 mg nalmefene by IM injection.	Each subject received both treatments, a single IN dose of 3 mg nalmefene HCl or a single IM dose of 1.0 mg nalmefene, by random assignment to one of 2 possible treatment sequences; a 4-day washout period separated treatments.	68 healthy subjects
PK Study 2 (NCT05219669; Crystal et al., 2024	Open-label, randomized, 6-sequence, 3-treatment, 3-period crossover study to assess the PK, safety, and tolerability of nalmefene following administration of three IN dosing regimens	Subjects were randomly assigned to 1 of 6 sequences (4 subjects per sequence) with each subject receiving all 3 regimens:<br>- 3 mg nalmefene HCl, one 0.1 mL spray in one nostril<br>- 6 mg nalmefene HCl, one 0.1 mL spray in each nostril<br>- 6 mg nalmefene HCl, two 0.1 mL sprays in one nostril<br>Each drug administration was separated by a washout period of 6 days.	24 healthy subjects
PD Study (NCT04828005; Ellison et al., 2024	Open-label, randomized, 2-period, 2-treatment, crossover study conducted in two parts (Part 1 and Part 2)<br>Part 1 (including Part 1 extension) was a pilot study to determine the relationship between remifentanil dose and suppression of CO_2-induced increases in minute ventilation in healthy volunteers with prior opioid exposure.<br>Part 2 aimed to evaluate the PD effects of IN nalmefene compared to IN naloxone in reversing remifentanil-induced suppression of CO_2-induced increases in minute ventilation in healthy volunteers with prior opioid exposure.	Part 1 (including Part 1 extension)<br>4 mg IN naloxone HCl, on Dose Day 1 and Dose Day 2, with a 4-day washout period between doses (Study Day 1 and Day 5).<br>Part 2:<br>Each subject received both treatments, a single IN dose of 3 mg nalmefene HCl or a single IN dose of 4.0 mg naloxone HCl, by random assignment to one of 2 possible treatment sequences; a 4-day washout period separated treatments.<br>On each dosing day, a remifentanil IV bolus dose (0.5 µg/kg) was administered at time 10 minutes, followed by a remifentanil infusion (rate: 0.175 µg/kg/min) which continued for the study duration.<br>At time 25 minutes, IN nalmefene or IN naloxone was administered.	Part 1:<br>7 healthy subjects with prior opioid exposure<br>Part 1 extension:<br>8 healthy subjects with prior opioid exposure<br>Part 2:<br>69 healthy subjects with prior opioid exposure

HCl, hydrochloride; IN, intranasal; IM, intramuscular; PD, pharmacodynamic; PK, pharmacokinetic.

Table S2 Population Pharmacokinetic Model Development for Intranasal Nalmefene and Intranasal Naloxone

<table>
<tr><th>Population PK Model</th><th>Data[1]</th><th>Methodology</th><th>Covariate Analysis</th></tr>
<tr><td>IN and IM Nalmefene</td><td>4401 quantifiable nalmefene plasma concentrations collected from 153 subjects enrolled in PK studies 1 & 2 and the PD study (Part 2)</td><td rowspan="2">The overall procedure for population PK model development included: 1) exploratory data analysis; 2) base structural model development; 3) evaluation of covariate effects; 4) final model refinement; and 5) model evaluation.
Results of the exploratory analyses were used to determine the appropriate functional form of the base structural model of drug plasma concentration versus time data.
Due to the variability in concentration profiles during IN absorption, several alternative absorption models were explored, including transit and sigmoid absorption, sequential zero- and first-order absorption, and parallel zero- and first-order absorption with and without lag times.
The same structural base model used for IN nalmefene was fit to IN naloxone PK data collected in the PD study. As PK sampling for naloxone only extended to 2 hours post dose, there was insufficient data to estimate all disposition parameters. Therefore, Q and Vp were fixed to population estimates from Yassen et al. (2007).
Interindividual variability was estimated as applicable assuming a log-normal distribution of individual PK parameters.
The adequacy of the final PK models was evaluated on the basis of the plausibility and precision of the parameter estimates, goodness-of-fit plots, and using a simulation-based, visual predictive check method to assess concordance between the observed data and model-based simulated data.
Population PK modeling was performed using the computer program NONMEM version 7.3. NONMEM analyses were performed on an Intel cluster with the Linux operating system.</td><td>A relative bioavailability parameter was included in nalmefene base model to simultaneously model IN and IM PK data. Additionally, a study effect on IN absorption was estimated based on exploratory data plots.
A systematic stepwise covariate screening examined the effects of the following stationary covariates based on baseline or screening assessments: age, body weight, body mass index, racial classification, sex, serum albumin, hematocrit, alanine aminotransferase, and aspartate aminotransferase. The forward selection followed by backward elimination approach was applied to covariate model building.</td></tr>
<tr><td>IN Naloxone</td><td>632 quantifiable naloxone plasma concentrations collected from 60 subjects enrolled in the PD study (Part 2)</td><td>No formal covariate analysis was conducted, but the effect of body weight on disposition parameters CL and V_c was assessed.</td></tr>
</table>

IN, intranasal; IM, intramuscular; PD, pharmacodynamic; PK, pharmacokinetic.
[1] Clinical studies are described in Table S1.

Table S3 Pharmacokinetic and Pharmacodynamic Parameters of Nalmefene, Naloxone and Remifentanil Used in the Translational Model

Compound	Parameter(s)	Estimate (Variability CV%)	Source
IN nalmefene	CL/F, V_c/F, Q/F, V_p/F, INKA, D2, INFK0, ALAG1, FR, σ^2	See Table 1	Clinical data (see Table S2)
	k_{e0} (sec^{-1})	0.001774	Assumed identical to k_{e0} for naloxone in Mann et al. (2022)
	k_{on} ($pM^{-n}sec^{-1}$)	2.06E-04	Cassel et al. (2005) and Mann et al. (2022)[1]
	k_{off} (sec^{-1})	1.35E-02	Cassel et al. (2005) and Mann et al. (2022)[2]
	n	0.86	Assumed identical to naloxone (Mann et al., 2022)
IN naloxone	CL/F, V_c/F, Q/F, V_p/F, KA, D2, FK0, ALAG1, σ^2	See Table 2	Clinical data (see Table S2)
	k_{e0} (sec^{-1})	0.001774	Mann et al. (2022)
	k_{on} ($pM^{-n}sec^{-1}$)	1.67E-04	
	k_{off} (sec^{-1})	3.96E-02	
	n	0.86	
IV remifentanil	CL (L/min)	2.58 (14.1%)	Eleveld et al. (2017)
	Q1 (L/min)	1.72 (23.7%)	
	Q2 (L/min)	0.124 (57.5%)	
	V1 (L)	5.81 (33.0%)	
	V2 (L)	8.82 (35.0%)	
	V3 (L)	5.03 (112%)	
	k_{e0} (sec^{-1})	0.0218	Olofsen et al. (2010)[3]
	k_{on} ($pM^{-n}sec^{-1}$)	8.08E-06	Mann et al. (2022)
	k_{off} (sec^{-1})	2.08E-03	
	n	0.70 (changed to 0.75 in the final model)	

CL, clearance of elimination; Q_n, clearance of distribution to compartment n; V_c, volume of central compartment; V_p, volume of peripheral compartment; V_n, volume of peripheral compartment n; k_{e0}, equilibration rate constant from plasma to effect compartment; k_{on}, association rate constant; koff, dissociation rate constant; n, steepness parameter of concentration-binding relationship.

[1] In Cassel et al. (2005), k_{on} values for nalmefene and naloxone were 58 and 47 $\mu mol^{-1}min^{-1}$, respectively. Nalmefene k_{on} value used for the simulations was obtained by multiplying the estimate of naloxone k_{on} from Mann et al. (2022) by the relative potency ratio "58/47" from Cassel et al.

[2] In Cassel et al. (2005), k_{off} values for nalmefene and naloxone were 0.29 and 0.85 min^{-1}, respectively. Nalmefene k_{off} value used for the simulations was obtained by multiplying the estimate of naloxone k_{off} from Mann et al. (2022) by the relative potency ratio "0.29/0.85" from Cassel et al.

[3] Calculated from the equilibration half-life estimate of 0.53 min.

Figure S1 Replication of Results from Mann et al. (2022): Effects of Delivering IM Naloxone in Different Volumes

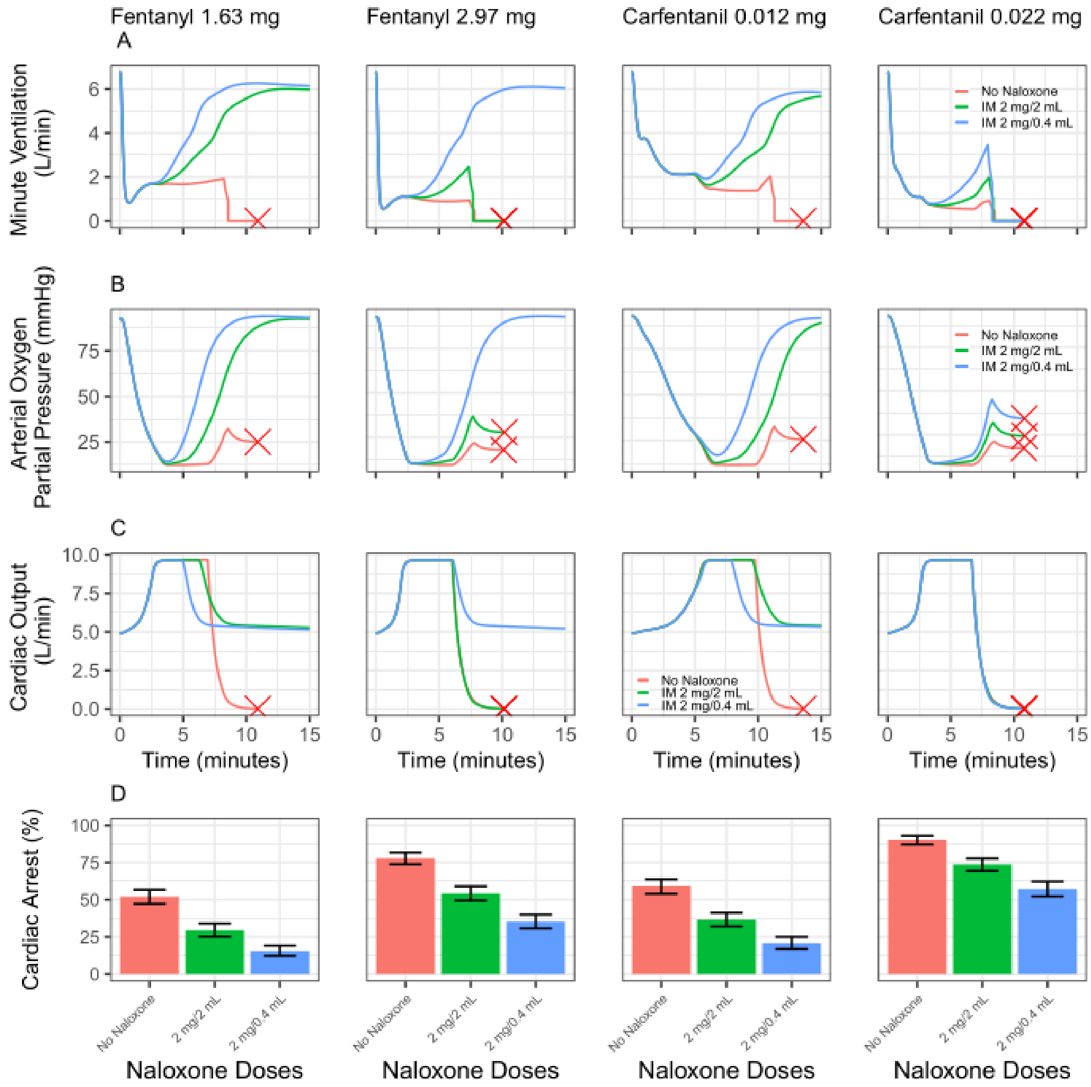

Model simulations were conducted to evaluate the effect of intravenous (IV) fentanyl (1.63 mg [Column 1] and 2.97 mg [Column 2]) and carfentanil (0.012 mg [Column 3] and 0.022 mg [Column 4]) on physiological variables and cardiac arrest in virtual chronic opioid users. The simulated values of minute ventilation (Panel A), arterial oxygen partial pressure (Panel B), and cardiac output (Panel C) are plotted versus time (minutes) for a typical virtual subject. The red X designates when a typical virtual subject had a complete cardiac arrest (that is, total blood flow near zero), which stopped the simulation. In Panel D, the simulated percentage of virtual subjects experiencing cardiac arrest is plotted versus naloxone doses. A single dose of intramuscular (IM) naloxone 2 mg/2 mL (green) is compared with a single IM dose of 2 mg naloxone in a volume of 0.4 mL (blue) and no opioid antagonist (red). Error bars represent the 2.5th and 97.5th percentiles after randomly sampling 400 out of the 2000 virtual chronic opioid users 2500 times. Mann et al. (2022) reported that a 2 mg/2 mL IM dose of naloxone resulted in a simulated incidence of cardiac arrest of 30% (28-31%) following a 1.63 mg IV dose of fentanyl. In this replication, the incidence of cardiac arrest under the same conditions was 29.5% (28-31%) as illustrated in Row D, left panel.

Figure S2 Visual Predictive Checks for the Nalmefene Population Pharmacokinetic Model Comparing Observed Plasma Concentrations to Model Predictions

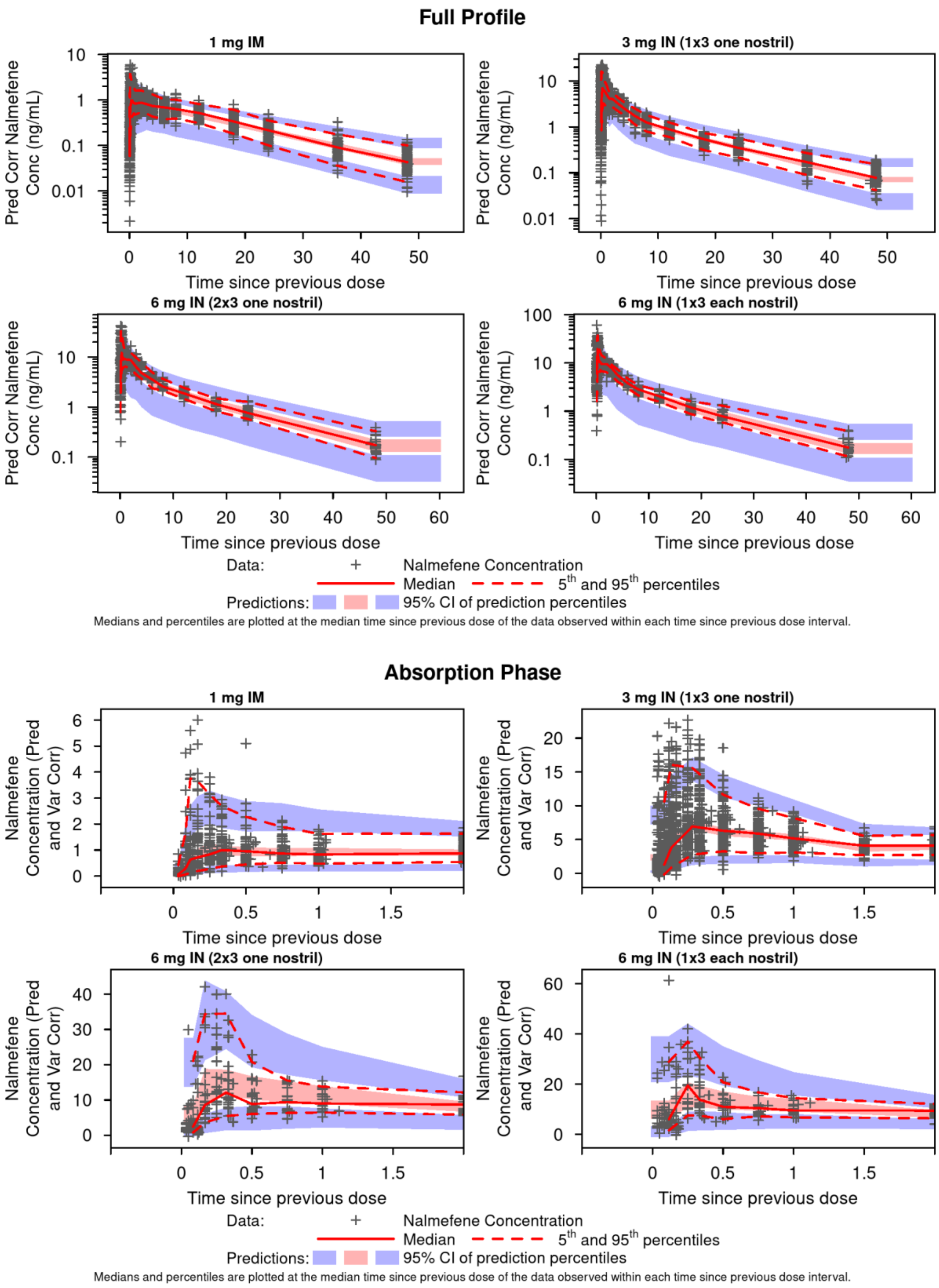

Figure S3 Visual Predictive Checks for the Naloxone Population Pharmacokinetic Model Comparing Observed Plasma Concentrations to Model Predictions

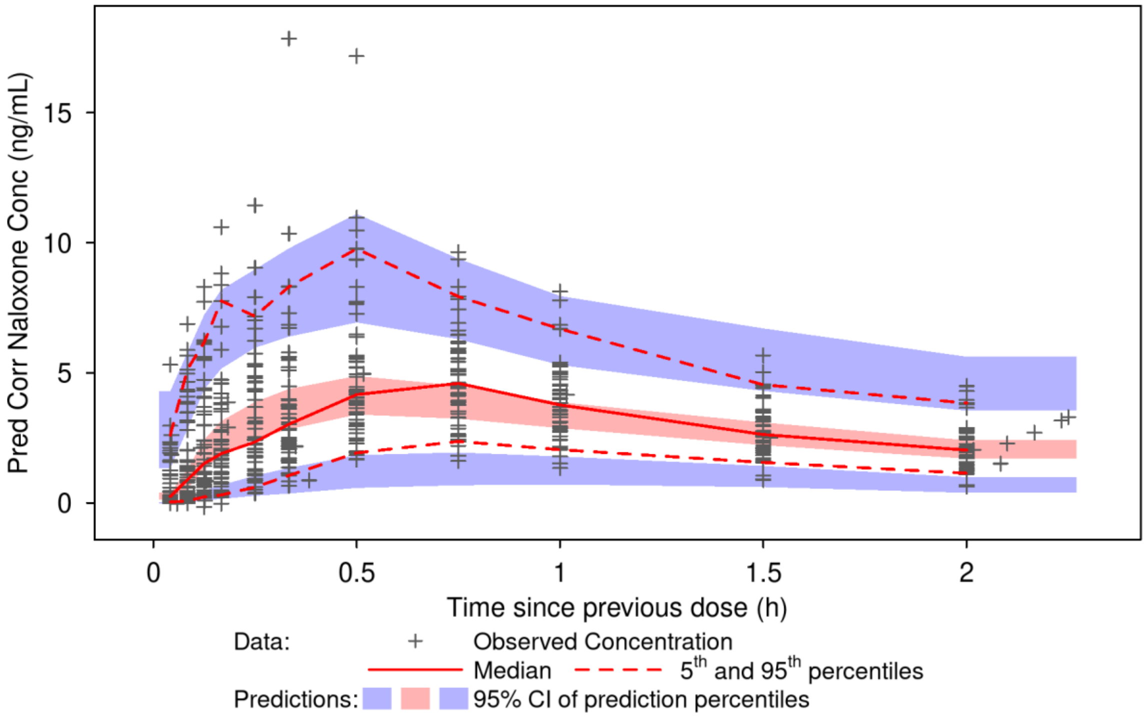

Medians and percentiles are plotted at the median time since previous dose of the data observed within each time since previous dose interval.

Supplement References

Cassel, J.A., Daubert, J.D. and DeHaven, R.N. (2005). [^{3}H] Alvimopan binding to the μ opioid receptor: comparative binding kinetics of opioid antagonists. European Journal of Pharmacology, 520(1-3), 29-36.

Eleveld, D.J., Proost, J.H., Vereecke, H., Absalom, A.R., Olofsen, E., Vuyk, J. and Struys, M.M. (2017). An allometric model of remifentanil pharmacokinetics and pharmacodynamics. Anesthesiology, 126(6), 1005-1018.

Mann, J., Samieegohar, M., Chaturbedi, A., Zirkle, J., Han, X., Ahmadi, S.F., Eshleman, A., Janowsky, A., Wolfrum, K., Swanson, T. and Bloom, S. (2022). Development of a translational model to assess the impact of opioid overdose and naloxone dosing on respiratory depression and cardiac arrest. Clinical Pharmacology & Therapeutics, 112(5), 1020-1032.

Olofsen, E., Boom, M., Nieuwenhuijs, D., Sarton, E., Teppema, L., Aarts, L. and Dahan, A. (2010). Modeling the non–steady state respiratory effects of remifentanil in awake and propofol-sedated healthy volunteers. Anesthesiology, 112(6), 1382-1395.

Yassen, A., Olofsen, E., van Dorp, E., Sarton, E., Teppema, L., Danhof, M. and Dahan, A. (2007). Mechanism-based pharmacokinetic-pharmacodynamic modelling of the reversal of buprenorphine-induced respiratory depression by naloxone: a study in healthy volunteers. Clinical Pharmacokinetics, 46, 965-980.
